# Real-world effectiveness of homologous and heterologous BNT162b2, CoronaVac, and AZD1222 booster vaccination against Delta and Omicron SARS-CoV-2 infection

**DOI:** 10.1080/22221751.2022.2072773

**Published:** 2022-05-23

**Authors:** Jing Lian Suah, Boon Hwa Tng, Peter Seah Keng Tok, Masliyana Husin, Thevesh Thevananthan, Kalaiarasu M. Peariasamy, Sheamini Sivasampu

**Affiliations:** a Institute for Clinical Research, National Institutes of Health, Ministry of Health Malaysia, Shah Alam, Malaysia; b Disease Control Division, Ministry of Health Malaysia, Putrajaya, Malaysia

**Keywords:** SARS-CoV-2, COVID-19 vaccines, booster, vaccine effectiveness, heterologous booster, homologous booster

## Abstract

Given emerging evidence of immune escape in the SARS-CoV-2 Omicron viral variant, and its dominance, effectiveness of heterologous and homologous boosting schedules commonly used in low-to-middle income countries needs to be re-evaluated. We conducted a test-negative design using consolidated national administrative data in Malaysia to compare the effectiveness of homologous and heterologous BNT162b2, CoronaVac, and AZD1222 booster vaccination against SARS-CoV-2 infection in predominant-Delta and predominant-Omicron periods. Across both periods, homologous CoronaVac and AZD1222 boosting demonstrated lower effectiveness than heterologous boosting for CoronaVac and AZD1222 primary vaccination recipients and homologous BNT162b2 boosting. Broadly, marginal effectiveness was smaller by 40–50 percentage points in the Omicron period than the Delta period. Without effective and accessible second-generation vaccines, heterologous boosting using BNT162b2 for inactivated and vectored primary vaccination recipients is preferred.

## Main

Immunogenic and real-world studies documented immune escape in Omicron amongst inactivated and mRNA primary and booster vaccine recipients [1–4]. Malaysia's national immunization programme administered a diverse portfolio of primarily BNT162b2, CoronaVac, and AZD1222 boosters [5] to address waning effectiveness of primary vaccination [6]. The rollout prioritized healthcare “frontliners,” and older individuals in an age-stepdown manner beginning 13 October 2021. The Government initially recommended BNT162b2 boosters. AZD1222 was subsequently included on 28 December 2021 for non-BNT162b2 primary vaccination recipients. CoronaVac boosters were available via private purchase, before being included in the national programme on 14 February 2022 for CoronaVac primary vaccination recipients. By 22 February 2022, 60.2% of the adult population received boosters. However, Omicron's dominance highlights the need to re-evaluate the effectiveness of heterologous and homologous boosting, especially CoronaVac and AZD1222.

To address this issue, we used a test-negative design derived from the vaccinated adult (aged ≥ 18) population from national administrative data: (i) register of COVID-19 vaccine recipients, (ii) register of supervised and approved reverse transcription polymerase chain reaction and antigen rapid tests conducted at all testing facilities, (iii) the COVID-19 confirmed cases line listing, (iv) register of healthcare “frontliners”, and (v) “contacts” (checked in via Malaysia's contact tracing application within a location-specific time window of another individual who tested positive for COVID-19 by end of day) identified by Malaysia's automated contact tracing system. Vaccination status is defined as “primary vaccinated” and “boosted” at 14 days post-dose two and three, respectively, at the point of testing.

Absent of adequate genomic surveillance, the Bai-Perron sequential structural break test [7] was applied to identify the predominant-Delta and Omicron periods by estimating trend breaks in daily SARS-CoV-2 infections (details in supplementary appendix). Marginal vaccine effectiveness (mVE) is calculated from the adjusted odds ratios (AOR) estimated using multivariable logistic regression using 100*(1-AOR), including as covariates (i) age, (ii) sex, (iii) presence of comorbidities, (iv) ethnicity (v) state of residence, (vi) whether primary vaccination was purchased privately, or received via the national programme, (vii) “frontliners” status (private healthcare worker, public healthcare worker, or otherwise), (viii) month of primary vaccination, (ix) baseline (before 27 October 2021) number of tests taken, and (X) baseline number of times flagged as “contact” (details in supplementary appendix).

All analyses were executed in Python 3.9, R 4.1.2, and EViews 11. Significance level of 5% was used for statistical inference.

The Bai-Perron sequential breakpoint test estimated 5 February 2022 as the start of the predominant-Omicron period. The study population comprises individuals aged 18 and above without prior infections who were boosted between 27 October 2021 and 4 February 2022, or primary vaccinated between 1 July 2021 and 30 September 2021. These correspond to 0–3 months post-boosted, and 4–6 months post-primary vaccinated, respectively. The latter reflects that effectiveness wanes 3 months post-primary vaccination [6], and the eligibility policy for booster doses.

Table 1 shows the estimated mVEs of booster vaccination relative to primary BNT162b2 vaccination (PP). In both predominant-Delta and predominant-Omicron periods, homologous CoronaVac and AZD1222 boosting (SSS and AAA) are less effective than heterologous boosting (SSP, SSA, and AAP) and homologous BNT162b2 boosting (PPP). The mVEs are smaller by 40–50 percentage points during the Omicron period than the Delta period, with homologous CoronaVac boosting registering the largest fall. Moreover, primary AZD1222 (AA) and CoronaVac (SS) vaccination recipients are of higher risk of SARS-CoV-2 infection than primary BNT162b2 vaccination (PP) recipients. A graphical representation can be found in the supplementary appendix.

**Table 1.** Marginal vaccine effectiveness against SARS-CoV-2 infection relative to BNT162b2 primary vaccination.

	Predominant-Delta (27 October 2021–4 February 2022)				Predominant-Omicron (5 February 2022–22 February 2022)			
Vaccination combination	Number of test-positives (%)	Number of test-negatives (%)	Unadjusted marginal vaccine effectiveness (95% CI)	Adjusted marginal vaccine effectiveness (95% CI)	Number of test-positives (%)	Number of test-negatives (%)	Unadjusted marginal vaccine effectiveness (95% CI)	Adjusted marginal vaccine effectiveness (95% CI)
Total	319,127 (16.6)	1,602,186 (83.4)	–	–	306,483 (32.1)	649,346 (67.9)	–	–
PPP	6828 (3.3)	202,393 (96.7)	87.84 (87.53, 88.14)	89.44 (89.17, 89.71)	31,202 (24.7)	95,131 (75.3)	57.60 (56.95, 58.24)	51.08 (50.29, 51.87)
SSA	622 (2.8)	21,992 (97.2)	89.81 (88.96, 90.59)	88.27 (87.29, 89.18)	2729 (20.9)	10,355 (79.1)	65.93 (64.43, 67.36)	49.05 (46.74, 51.26)
SSP	22,044 (4.4)	482,314 (95.6)	83.53 (83.28, 83.77)	85.11 (84.87, 85.34)	68,961 (23.4)	225,554 (76.6)	60.47 (60.00, 60.94)	47.64 (46.95, 48.32)
SSS	3965 (5.7)	65,776 (94.3)	78.27 (77.55, 78.97)	82.25 (81.64, 82.85)	13,931 (27.2)	37,304 (72.8)	51.72 (50.69, 52.73)	33.42 (31.85, 34.95)
AAP	1944 (3.4)	54,829 (96.6)	87.22 (86.62, 87.79)	86.59 (85.95, 87.19)	7155 (21.1)	26,689 (78.9)	65.34 (64.38, 66.28)	52.96 (51.59, 54.29)
AAA	3164 (5.5)	54,805 (94.5)	79.19 (78.42, 79.94)	76.08 (75.17, 76.96)	11,447 (27.7)	29,935 (72.3)	50.56 (49.41, 51.69)	30.14 (28.39, 31.84)
SS	138,827 (34.8)	259,764 (65.2)	−92.62 (−94.40, −90.85)	−100.87 (−102.88, −98.89)	58,852 (42.7)	79,101 (57.3)	3.81 (2.51, 5.10)	−14.74 (−16.39, −13.11)
AA	25,784 (37.8)	42,418 (62.2)	−119.08 (−122.79, −115.43)	−158.02 (−162.72, −153.4)	11,874 (43.3)	15,566 (56.7)	1.38 (−1.14, 3.84)	−19.72 (−22.93, −16.60)
PP	115,949 (21.7)	417,895 (78.3)	Reference	Reference	100,332 (43.6)	129,711 (56.4)	Reference	Reference

Note: PPP: 3× BNT162b2; SSA: 2× CoronaVac+AZD1222; SSP: 2× CoronaVac+BNT162b2; SSS: 3× CoronaVac; AAP: 2× AZD1222+BNT162b2; AAA: 3× AZD1222; SS: 2× CoronaVac; AA: 2× AZD1222; PP: 2× BNT162b2 (reference group).

## Discussion

This study has three strengths. Firstly, to the best of our knowledge, this is the first comparison of homologous and heterologous booster effectiveness for CoronaVac and AZD1222 primary vaccination recipients under Delta and Omicron dominance in a low-to-middle income country (LMIC). Secondly, this study considers a diverse set of commonly used homologous and heterologous vaccinations. Thirdly, granular national administrative data underpinned the analysis.

The main limitation is the potential undermeasurement of SARS-CoV-2 infections due to the use of self-test kits. Nevertheless, supervised test rates were higher during the Delta and Omicron periods (averaged 362 and 715 per 100,000, respectively) than the rest of 2021 (302 per 100,000). While Malaysia adopted a symptomatic testing strategy, asymptomatic SARS-CoV-2 infections may be included as there were no formal barriers to access supervised testing. Due to the rapid rollout of boosters, and short follow-up duration, potential waning of effectiveness could not be analysed. Importantly, while a wide range of confounders were adjusted for, including exposure risk, health-seeking behaviour, underlying conditions, and batch effects, residual confounding may still be present in observational studies such as this.

First-generation boosters were less effective against Omicron than Delta SARS-CoV-2 infection. In both periods, heterologous boosting was more effective than homologous boosting for AZD1222 and CoronaVac primary vaccination recipients. Our analysis complements pre-Omicron findings that homologous boosting with AZD1222 in the United Kingdom [8] and CoronaVac in Brazil [9], and Omicron-specific findings in Hong Kong [1] that homologous CoronaVac boosting generated lower neutralizing antibodies than mRNA boosters. Pre-Omicron evidence from Chile [10] observed lower effectiveness for homologous than heterologous boosting in CoronaVac primary vaccination recipients. Hence, absent of second-generation vaccines during the predominant-Omicron period, heterologous boosting using BNT162b2 for inactivated and vectored primary vaccination recipients is preferred.

## Ethical consideration

This is part of The Real-World Evaluation of COVID-19 Vaccines under the Malaysia National COVID-19 Immunization Programme (RECoVaM) study registered in the National Medical Research Register (NMRR-21-1660-60697). This study was granted ethical approval by the Medical Research and Ethics Committee (MREC), Ministry of Health, Malaysia.

## Supplementary Material

Supplemental Material
