# Supplementary material for: Real-world effectiveness of homologous and heterologous BNT162b2, CoronaVac, and AZD1222 booster vaccination against Delta and Omicron SARS-CoV-2 infection
Source: Emerg Microbes Infect. 2022 May 23;11(1):1343–5. doi: 10.1080/22221751.2022.2072773 (PMC9132393; doi:10.1080/22221751.2022.2072773)
Supplement: Supplemental Material [file TEMI_A_2072773_SM4297.docx]

# Supplementary Appendix for “Real-World Effectiveness of Homologous and Heterologous BNT162b2, CoronaVac, and AZD1222 Booster Vaccination Against Delta and Omicron SARS-CoV-2 Infection”

**Bai-Perron Sequential Breakpoint Test**

The Bai-Perron sequential breakpoint test estimates the location of, and formally tests for the presence of, multiple unknown trend-breaks in a time series. This specific test, along with other variants, is introduced in Bai and Perron (2003). Briefly, the sequential breakpoint test has three steps. Firstly, the time series of interest, which is the daily number of SARS-CoV-2 infections based on all supervised tests conducted in Malaysia, with positive tests within 90 days since another positive test ignored according to individuals, is regressed on a constant (equation 1). The time series is expressed as a 7-day moving average to smooth out daily noise. The parameters are estimated using ordinary least squares. Secondly, the error term is modelled as a function of time (equation 2), from specific breakpoints are uncovered by minimising the sum of squared errors (equation 3). Thirdly, these estimated breakpoints are tested for statistical significance in sequence (equation 4) up to a total of 5 possible breakpoints.

| $y_{t}=\alpha+\epsilon_{t}$ | (1) |
| --- | --- |
| $y_{t}=\alpha+\epsilon_{t}\left( t=t_{j-1}+1, \ldots,t_{j};j=1, \ldots,m+1 \right)$ | (2) |
| $\boldsymbol{t}_{\boldsymbol{break}}\boldsymbol{\equiv}\min\left( \hat{\boldsymbol{\epsilon}}\boldsymbol{'}\hat{\boldsymbol{\epsilon}} \right)$ | (3) |
| $\boldsymbol{l+1}vs.\boldsymbol{l}breaks, for 0\leq\boldsymbol{l}\leq5$ | (4) |

**Figure S1** shows the time series of daily number of SARS-CoV-2 infections indicated by supervised RT-PCR and RTK-Ag tests in Malaysia, as well as the breakpoint estimated by the Bai-Perron sequential breakpoint test, which is taken as the start of the predominantly-Omicron period. We estimated only one breakpoint on 5 February 2022 using a significance level of 5%, with a narrow 95% confidence interval of 3 February 2022 to 6 February 2022. **Table S1** summarises the sequential tests, and associated Bai-Perron critical values.

**Figure S1:** **SARS-CoV-2 Infections and Bai-Perron Breakpoint between 27 October 2021 and 20 February 2022; Red bands correspond to the estimated 95% confidence intervals**


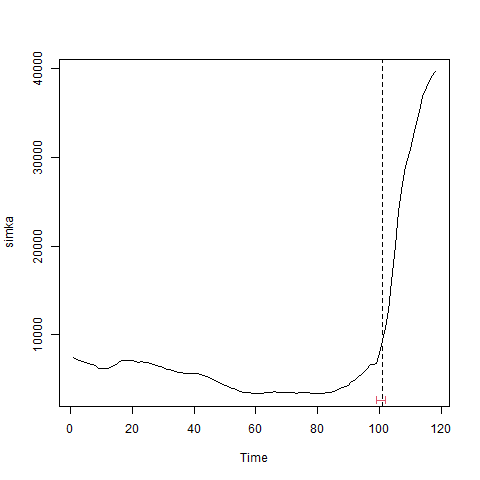


**Table S1: Bai-Perron Sequential Break Test**

| **Sequential F-statistic determined breaks: 1** | | | |
| --- | --- | --- | --- |
| **Break test** | **F-statistic** | **Scaled F-statistic** | **Bai-Perron critical values** |
| **0 vs 1 *** | 587.71 | 587.71 | **8.58** |
| **1 vs 2** | 8.97 | 8.97 | **10.13** |
|  | | | |
| **Break dates: 5 February 2022** | | | |

*Significant at the 0.05 level

**Test-Negative Design and Multivariable Logistic Regression**

**Figure S2** details the exclusion and inclusion criteria of the test-negative design. The analysis includes the first positive test, both RT-PCR and RTK-Ag tests, for individuals with records of positive tests. For individuals who never tested positive, only the first negative test is included. This step attempts to minimise the dependency of individual probabilities of exposure to SARS-CoV-2 on when vaccination occurred. Amongst 1,921,313 eligible tested individuals during the predominant-Delta period (27 October 2021 to 4 February 2022), 319,127 tested positive, and 1,602,186 tested negative. Amongst 955,829 eligible tested individuals during the predominant-Omicron period (5 February 2022 to 22 February 2022), 305,483 tested positive, and 649,346 tested negative.

**Figure S2: Inclusion and Exclusion Rules of the Study Population**

**
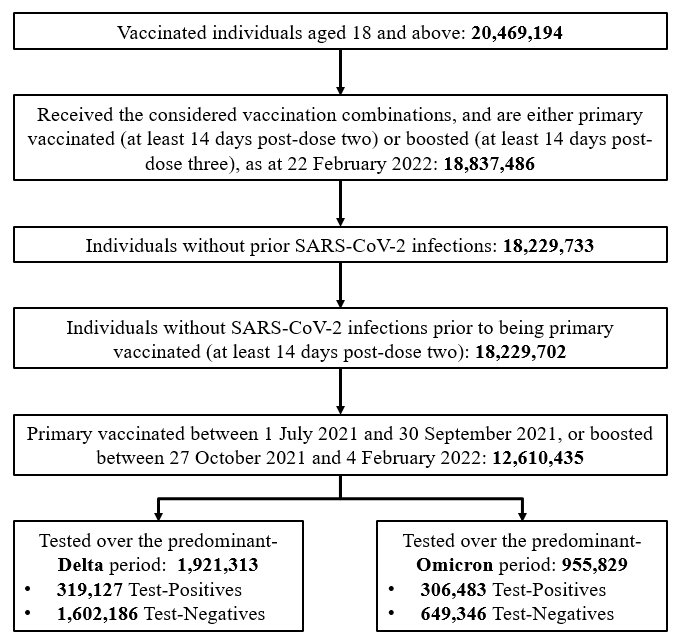
**

Using a multivariable logistic regression approach (equation 5), the set of **marginal vaccine effectiveness (mVE)** are estimated separately for the predominant-Delta period, and the predominant-Omicron period. However, the vector of all vaccine combinations (**V**), and the vector of controls (**X**) are included in each estimation iteration. ϵ refers to the error term. Vector **X** contains (i) age as a continuous variable, (ii) sex (male or female), (iii) presence of comorbidities (yes or no), (iv) ethnicity (Malay, Chinese, Indian, Sarawakian Natives, Sabahan Natives, Other Natives, Indigenous People, and Other Ethnicities) (v) state of residence (14 states, with Selangor, Kuala Lumpur, and Putrajaya treated as a single state, reflecting the Greater Klang Valley metropolitan area), (vi) whether primary vaccination was purchased privately, or received via the national programme, (vii) ‘frontliners’ status (private healthcare worker, public healthcare worker, or otherwise), (viii) month of primary vaccination, (ix) baseline (before 27 October 2021) number of tests taken, and (x) baseline number of times flagged as ‘contact’ (checked in via Malaysia’s contact tracing application within a location-specific time window of another individual who tested positive for COVID-19 by end of day). Specifically, parameters **β** in equation 5 were estimated using maximum likelihood estimation (MLE), using the Newton-conjugate gradient numerical optimisation approach. The **mVEs** for vaccine type v is the calculated from the estimated parameters **β** as in equation 6.

| $Y=\Lambda\left( \alpha+\boldsymbol{V\beta}+\boldsymbol{X\lambda}+\epsilon\right)$ | (5) |
| --- | --- |
| $mVE_{v}=100*\left( 1-\exp\left( \hat{\beta} \right) \right)$ | (6) |

**Additional Results**

**Table S2: Baseline Characteristics of Study Population for the Predominantly Delta Period (27 October 2021 to 4 February 2022)**

|  | **2x BNT162b2** | **2x AZD1222** | **2x CoronaVac** | **3x AZD1222** | **2x AZD1222 + BNT162b2** | **3x BNT162b2** | **2x CoronaVac + AZD1222** | **2x CoronaVac + BNT162b2** | **3x CoronaVac** |
| --- | --- | --- | --- | --- | --- | --- | --- | --- | --- |
| **Number of Individuals (%)** | **533844** | **68202** | **398591** | **57969** | **56773** | **209221** | **22614** | **504358** | **69741** |
| **SARS-CoV-2 Infection** |  |  |  |  |  |  |  |  |  |
| Yes | 115949 (21.7) | 25784 (37.8) | 138827 (34.8) | 3164 (5.5) | 1944 (3.4) | 6828 (3.3) | 622 (2.8) | 22044 (4.4) | 3965 (5.7) |
| No | 417895 (78.3) | 42418 (62.2) | 259764 (65.2) | 54805 (94.5) | 54829 (96.6) | 202393 (96.7) | 21992 (97.2) | 482314 (95.6) | 65776 (94.3) |
| **Age** |  |  |  |  |  |  |  |  |  |
| 18-39 | 351880 (65.9) | 44647 (65.5) | 268997 (67.5) | 38669 (66.7) | 22000 (38.8) | 89882 (43) | 16827 (74.4) | 272462 (54) | 33708 (48.3) |
| 40-59 | 124712 (23.4) | 13242 (19.4) | 90367 (22.7) | 13089 (22.6) | 22078 (38.9) | 84014 (40.2) | 4875 (21.6) | 180330 (35.8) | 25967 (37.2) |
| 60+ | 57252 (10.7) | 10313 (15.1) | 39227 (9.8) | 6211 (10.7) | 12695 (22.4) | 35325 (16.9) | 912 (4) | 51566 (10.2) | 10066 (14.4) |
| **Presence of Comorbidities** |  |  |  |  |  |  |  |  |  |
| Yes | 194867 (36.5) | 21280 (31.2) | 113532 (28.5) | 16660 (28.7) | 22244 (39.2) | 93770 (44.8) | 4798 (21.2) | 158478 (31.4) | 22511 (32.3) |
| No | 338977 (63.5) | 46922 (68.8) | 285059 (71.5) | 41309 (71.3) | 34529 (60.8) | 115451 (55.2) | 17816 (78.8) | 345880 (68.6) | 47230 (67.7) |
| **Sex** |  |  |  |  |  |  |  |  |  |
| Female | 291378 (54.6) | 33804 (49.6) | 172963 (43.4) | 26072 (45) | 26571 (46.8) | 97672 (46.7) | 8568 (37.9) | 214884 (42.6) | 30908 (44.3) |
| Male | 242466 (45.4) | 34398 (50.4) | 225628 (56.6) | 31897 (55) | 30202 (53.2) | 111549 (53.3) | 14046 (62.1) | 289474 (57.4) | 38833 (55.7) |
| **Ethnicity** |  |  |  |  |  |  |  |  |  |
| Malay | 359616 (67.4) | 44205 (64.8) | 240178 (60.3) | 27932 (48.2) | 25723 (45.3) | 106426 (50.9) | 12743 (56.4) | 261573 (51.9) | 8000 (11.5) |
| Chinese | 55499 (10.4) | 14731 (21.6) | 51929 (13) | 23943 (41.3) | 22985 (40.5) | 60806 (29.1) | 3555 (15.7) | 122657 (24.3) | 53101 (76.1) |
| Indian | 36253 (6.8) | 3817 (5.6) | 23517 (5.9) | 3206 (5.5) | 3538 (6.2) | 16382 (7.8) | 1750 (7.7) | 35372 (7) | 1921 (2.8) |
| BMP Sarawak | 6017 (1.1) | 612 (0.9) | 13658 (3.4) | 332 (0.6) | 788 (1.4) | 4847 (2.3) | 163 (0.7) | 26541 (5.3) | 1668 (2.4) |
| BMP Sabah | 48307 (9) | 315 (0.5) | 19808 (5) | 293 (0.5) | 193 (0.3) | 10705 (5.1) | 350 (1.5) | 11810 (2.3) | 1690 (2.4) |
| BMP Others | 122 (0) | 1 (0) | 155 (0) | 0 (0) | 0 (0) | 72 (0) | 0 (0) | 67 (0) | 3 (0) |
| Orang Asli | 1334 (0.2) | 25 (0) | 1009 (0.3) | 14 (0) | 17 (0) | 273 (0.1) | 55 (0.2) | 818 (0.2) | 68 (0.1) |
| Others | 26696 (5) | 4496 (6.6) | 48337 (12.1) | 2249 (3.9) | 3529 (6.2) | 9710 (4.6) | 3998 (17.7) | 45520 (9) | 3290 (4.7) |
| **Frontliner Status** |  |  |  |  |  |  |  |  |  |
| General Population | 525511 (98.4) | 66937 (98.1) | 394963 (99.1) | 56776 (97.9) | 55012 (96.9) | 204150 (97.6) | 22233 (98.3) | 496517 (98.4) | 68775 (98.6) |
| Public Healthcare | 2080 (0.4) | 7 (0) | 159 (0) | 0 (0) | 17 (0) | 939 (0.4) | 3 (0) | 144 (0) | 29 (0) |
| Private Healthcare | 6253 (1.2) | 1258 (1.8) | 3469 (0.9) | 1193 (2.1) | 1744 (3.1) | 4132 (2) | 378 (1.7) | 7697 (1.5) | 937 (1.3) |
| **Baseline Number of Times Flagged As 'Contact'** |  |  |  |  |  |  |  |  |  |
| 0 | 447009 (83.7) | 53112 (77.9) | 318614 (79.9) | 44113 (76.1) | 43959 (77.4) | 169373 (81) | 16498 (73) | 382513 (75.8) | 55696 (79.9) |
| 1 | 67032 (12.6) | 11617 (17) | 57159 (14.3) | 10729 (18.5) | 9942 (17.5) | 30263 (14.5) | 4181 (18.5) | 86682 (17.2) | 10636 (15.3) |
| 2 to 4 | 18964 (3.6) | 3375 (4.9) | 21173 (5.3) | 3009 (5.2) | 2775 (4.9) | 9145 (4.4) | 1742 (7.7) | 32451 (6.4) | 3192 (4.6) |
| 5 to 9 | 791 (0.1) | 94 (0.1) | 1547 (0.4) | 112 (0.2) | 91 (0.2) | 415 (0.2) | 178 (0.8) | 2540 (0.5) | 197 (0.3) |
| 10+ | 48 (0) | 4 (0) | 98 (0) | 6 (0) | 6 (0) | 25 (0) | 15 (0.1) | 172 (0) | 20 (0) |
| **Baseline Number of Supervised Tests Taken** |  |  |  |  |  |  |  |  |  |
| 0 | 356160 (66.7) | 46220 (67.8) | 251024 (63) | 35426 (61.1) | 36103 (63.6) | 121680 (58.2) | 11086 (49) | 269114 (53.4) | 43249 (62) |
| 1 | 87706 (16.4) | 11661 (17.1) | 66904 (16.8) | 10537 (18.2) | 10292 (18.1) | 35404 (16.9) | 4110 (18.2) | 84291 (16.7) | 11876 (17) |
| 2 to 4 | 67164 (12.6) | 7988 (11.7) | 56118 (14.1) | 8336 (14.4) | 7738 (13.6) | 34069 (16.3) | 4295 (19) | 91189 (18.1) | 10108 (14.5) |
| 5 to 9 | 18599 (3.5) | 1868 (2.7) | 19482 (4.9) | 2850 (4.9) | 2112 (3.7) | 14271 (6.8) | 2264 (10) | 47736 (9.5) | 3646 (5.2) |
| 10+ | 4215 (0.8) | 465 (0.7) | 5063 (1.3) | 820 (1.4) | 528 (0.9) | 3797 (1.8) | 859 (3.8) | 12028 (2.4) | 862 (1.2) |
| **Mode of Obtaining Primary Vaccination** |  |  |  |  |  |  |  |  |  |
| For Free Via PICK | 533843 (100) | 68202 (100) | 389768 (97.8) | 57969 (100) | 56773 (100) | 209221 (100) | 22467 (99.3) | 497699 (98.7) | 66035 (94.7) |
| Private Purchase | 1 (0) | 0 (0) | 8823 (2.2) | 0 (0) | 0 (0) | 0 (0) | 147 (0.7) | 6659 (1.3) | 3706 (5.3) |

PICK: Program Imunisasi COVID-19 Kebangsaan (National COVID-19 Immunisation Programme)

**Table S3: Baseline Characteristics of Study Population for the Predominantly Omicron Period (5 February 2022 to 22 February 2022)**

|  | **2x BNT162b2** | **2x AZD1222** | **2x CoronaVac** | **3x AZD1222** | **2x AZD1222 + BNT162b2** | **3x BNT162b2** | **2x CoronaVac + AZD1222** | **2x CoronaVac + BNT162b2** | **3x CoronaVac** |
| --- | --- | --- | --- | --- | --- | --- | --- | --- | --- |
| **Number of Individuals (%)** | **230043** | **27440** | **137953** | **41382** | **33844** | **126333** | **13084** | **294515** | **51235** |
| **SARS-CoV-2 Infection** |  |  |  |  |  |  |  |  |  |
| Yes | 100332 (43.6) | 11874 (43.3) | 58852 (42.7) | 11447 (27.7) | 7155 (21.1) | 31202 (24.7) | 2729 (20.9) | 68961 (23.4) | 13931 (27.2) |
| No | 129711 (56.4) | 15566 (56.7) | 79101 (57.3) | 29935 (72.3) | 26689 (78.9) | 95131 (75.3) | 10355 (79.1) | 225554 (76.6) | 37304 (72.8) |
| **Age** |  |  |  |  |  |  |  |  |  |
| 18-39 | 163095 (70.9) | 20715 (75.5) | 103488 (75) | 29004 (70.1) | 14408 (42.6) | 56151 (44.4) | 10358 (79.2) | 165798 (56.3) | 27646 (54) |
| 40-59 | 50352 (21.9) | 3992 (14.5) | 25645 (18.6) | 8673 (21) | 12432 (36.7) | 51434 (40.7) | 2430 (18.6) | 103595 (35.2) | 17959 (35.1) |
| 60+ | 16596 (7.2) | 2733 (10) | 8820 (6.4) | 3705 (9) | 7004 (20.7) | 18748 (14.8) | 296 (2.3) | 25122 (8.5) | 5630 (11) |
| **Presence of Comorbidities** |  |  |  |  |  |  |  |  |  |
| Yes | 74897 (32.6) | 7243 (26.4) | 34467 (25) | 11106 (26.8) | 12635 (37.3) | 53813 (42.6) | 2421 (18.5) | 86698 (29.4) | 14854 (29) |
| No | 155146 (67.4) | 20197 (73.6) | 103486 (75) | 30276 (73.2) | 21209 (62.7) | 72520 (57.4) | 10663 (81.5) | 207817 (70.6) | 36381 (71) |
| **Sex** |  |  |  |  |  |  |  |  |  |
| Female | 121779 (52.9) | 13983 (51) | 60749 (44) | 20005 (48.3) | 16576 (49) | 61941 (49) | 5489 (42) | 134914 (45.8) | 24632 (48.1) |
| Male | 108264 (47.1) | 13457 (49) | 77204 (56) | 21377 (51.7) | 17268 (51) | 64392 (51) | 7595 (58) | 159601 (54.2) | 26603 (51.9) |
| **Ethnicity** |  |  |  |  |  |  |  |  |  |
| Malay | 145306 (63.2) | 18671 (68) | 88458 (64.1) | 18493 (44.7) | 14589 (43.1) | 58718 (46.5) | 6976 (53.3) | 145338 (49.3) | 4608 (9) |
| Chinese | 33535 (14.6) | 6407 (23.3) | 20055 (14.5) | 19844 (48) | 16038 (47.4) | 46259 (36.6) | 3136 (24) | 95612 (32.5) | 41857 (81.7) |
| Indian | 16021 (7) | 1249 (4.6) | 9467 (6.9) | 1741 (4.2) | 1563 (4.6) | 8480 (6.7) | 986 (7.5) | 19685 (6.7) | 1016 (2) |
| BMP Sarawak | 1477 (0.6) | 113 (0.4) | 2071 (1.5) | 168 (0.4) | 260 (0.8) | 1573 (1.2) | 84 (0.6) | 7976 (2.7) | 550 (1.1) |
| BMP Sabah | 25263 (11) | 121 (0.4) | 8785 (6.4) | 152 (0.4) | 80 (0.2) | 6709 (5.3) | 159 (1.2) | 7127 (2.4) | 1569 (3.1) |
| BMP Others | 42 (0) | 0 (0) | 64 (0) | 0 (0) | 0 (0) | 40 (0) | 0 (0) | 37 (0) | 3 (0) |
| Orang Asli | 327 (0.1) | 4 (0) | 180 (0.1) | 4 (0) | 9 (0) | 80 (0.1) | 23 (0.2) | 294 (0.1) | 30 (0.1) |
| Others | 8072 (3.5) | 875 (3.2) | 8873 (6.4) | 980 (2.4) | 1305 (3.9) | 4474 (3.5) | 1720 (13.1) | 18446 (6.3) | 1602 (3.1) |
| **Frontliner Status** |  |  |  |  |  |  |  |  |  |
| General Population | 225790 (98.2) | 26839 (97.8) | 136476 (98.9) | 40531 (97.9) | 32624 (96.4) | 122939 (97.3) | 12828 (98) | 289330 (98.2) | 50521 (98.6) |
| Public Healthcare | 1118 (0.5) | 3 (0) | 91 (0.1) | 0 (0) | 9 (0) | 665 (0.5) | 2 (0) | 91 (0) | 37 (0.1) |
| Private Healthcare | 3135 (1.4) | 598 (2.2) | 1386 (1) | 851 (2.1) | 1211 (3.6) | 2729 (2.2) | 254 (1.9) | 5094 (1.7) | 677 (1.3) |
| **Baseline Number of Times Flagged As 'Contact'** |  |  |  |  |  |  |  |  |  |
| 0 | 187217 (81.4) | 21266 (77.5) | 108229 (78.5) | 31469 (76) | 26571 (78.5) | 101896 (80.7) | 9411 (71.9) | 224134 (76.1) | 41383 (80.8) |
| 1 | 32571 (14.2) | 4811 (17.5) | 21648 (15.7) | 7626 (18.4) | 5706 (16.9) | 18538 (14.7) | 2527 (19.3) | 50753 (17.2) | 7537 (14.7) |
| 2 to 4 | 9840 (4.3) | 1327 (4.8) | 7546 (5.5) | 2199 (5.3) | 1511 (4.5) | 5647 (4.5) | 1043 (8) | 18151 (6.2) | 2176 (4.2) |
| 5 to 9 | 396 (0.2) | 35 (0.1) | 502 (0.4) | 84 (0.2) | 53 (0.2) | 236 (0.2) | 99 (0.8) | 1391 (0.5) | 132 (0.3) |
| 10+ | 19 (0) | 1 (0) | 28 (0) | 4 (0) | 3 (0) | 16 (0) | 4 (0) | 86 (0) | 7 (0) |
| **Baseline Number of Supervised Tests Taken** |  |  |  |  |  |  |  |  |  |
| 0 | 147342 (64) | 17896 (65.2) | 80841 (58.6) | 26765 (64.7) | 22378 (66.1) | 75526 (59.8) | 6411 (49) | 160393 (54.5) | 34400 (67.1) |
| 1 | 38795 (16.9) | 4867 (17.7) | 23007 (16.7) | 6990 (16.9) | 5550 (16.4) | 19676 (15.6) | 2103 (16.1) | 45154 (15.3) | 7973 (15.6) |
| 2 to 4 | 30631 (13.3) | 3427 (12.5) | 21045 (15.3) | 5103 (12.3) | 4148 (12.3) | 18653 (14.8) | 2353 (18) | 48809 (16.6) | 6011 (11.7) |
| 5 to 9 | 10584 (4.6) | 951 (3.5) | 9945 (7.2) | 1925 (4.7) | 1374 (4.1) | 9585 (7.6) | 1583 (12.1) | 31314 (10.6) | 2233 (4.4) |
| 10+ | 2691 (1.2) | 299 (1.1) | 3115 (2.3) | 599 (1.4) | 394 (1.2) | 2893 (2.3) | 634 (4.8) | 8845 (3) | 618 (1.2) |
| **Mode of Obtaining Primary Vaccination** |  |  |  |  |  |  |  |  |  |
| For Free Via PICK | 230043 (100) | 27440 (100) | 134361 (97.4) | 41382 (100) | 33844 (100) | 126333 (100) | 12983 (99.2) | 289726 (98.4) | 47660 (93) |
| Private Purchase | 0 (0) | 0 (0) | 3592 (2.6) | 0 (0) | 0 (0) | 0 (0) | 101 (0.8) | 4789 (1.6) | 3575 (7) |

PICK: Program Imunisasi COVID-19 Kebangsaan (National COVID-19 Immunisation Programme)

**Figure S3: Adjusted Marginal Vaccine Effectiveness Against SARS-CoV-2 Infection Relative to BNT162b2 Primary Vaccination**

**
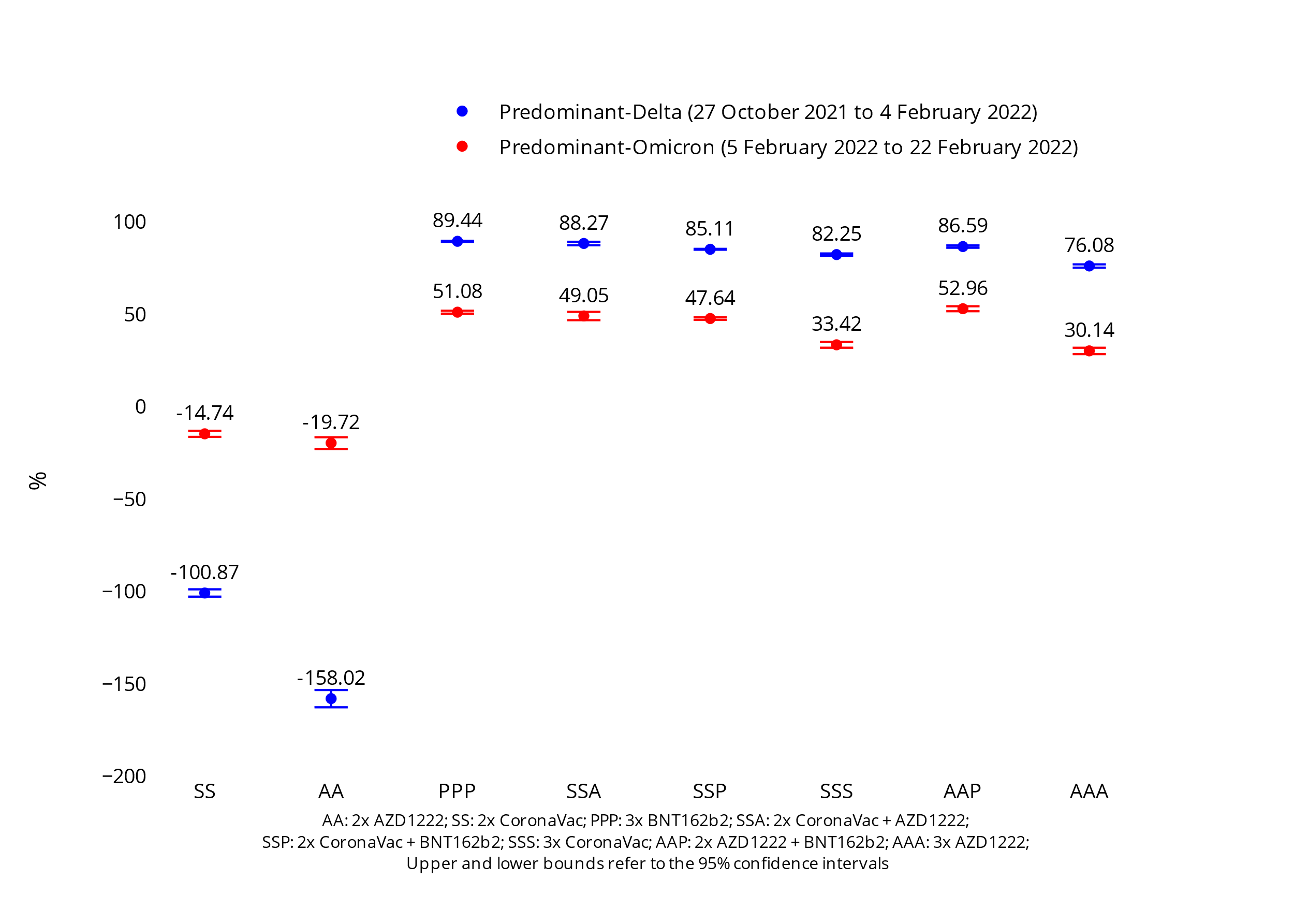
**
